# 452. Long-Term Risk of Arrhythmias After Infective Endocarditis: A Global Propensity-Matched Cohort Study

**DOI:** 10.1093/ofid/ofaf695.151

**Published:** 2026-01-11

**Authors:** Siddartha Guru, Paddy Ssentongo, Nadim Jaafar, Chen Song

**Affiliations:** Penn State Health Milton S. Hershey Medical Center, Hummelstown, PA; Penn State Health Milton S. Hershey Medical Center, Hummelstown, PA; Greater Baltimore Medical Center, Towson, Maryland; Penn State Health Milton S. Hershey Medical Center, Hummelstown, PA

## Abstract

**Background:**

Infective endocarditis (IE) is a serious condition with potential long-term cardiac complications, yet the risk of arrhythmia development following IE is not well established. This study aimed to quantify the long-term risk of clinically significant arrhythmias among IE survivors.

Fig 1

**Figure:**
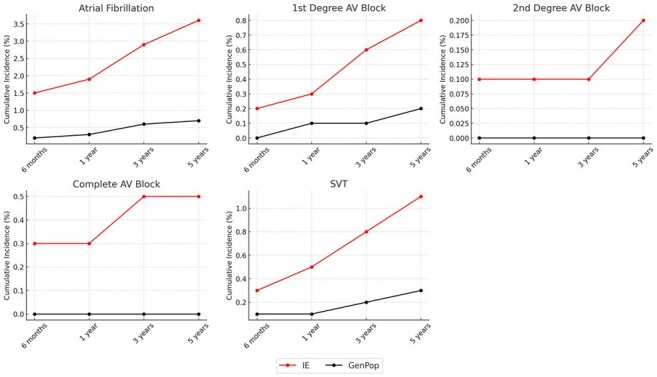

Cumulative Risk of Arrhythmias Following Infective Endocarditis Compared to the General Population. Figure displays the cumulative incidence of five arrhythmia subtypes—atrial fibrillation/flutter (AF), first-degree atrioventricular (AV) block, second-degree AV block, complete AV block, and supraventricular tachycardia (SVT)—over 6 months, 1 year, 3 years, and 5 years following index diagnosis of infective endocarditis (IE). Red lines represent the IE cohort; black lines represent a propensity-matched general population cohort. All arrhythmia subtypes demonstrated significantly increased risk in the IE cohort over time, with divergence in event rates evident as early as 6 months and persisting through 5 years.

**Methods:**

We conducted a retrospective, propensity score–matched cohort study using the TriNetX Global Collaborative Network, comprising electronic health records from 144 health care organizations. Adults aged ≥18 years diagnosed with IE (ICD-10 I33.0) without prior arrhythmias or structural heart disease were identified and matched 1:1 to a general population cohort with no prior cardiac disease. Matching variables included age, sex, hypertension, diabetes, obesity, tobacco use, and substance use disorders. Outcomes were assessed over 5 years from the index diagnosis using Kaplan-Meier survival analysis and Cox proportional hazards modeling. Primary outcomes included incident atrial fibrillation/flutter (AF), ventricular fibrillation/flutter (VF), atrioventricular (AV) block (first, second, and complete), sick sinus syndrome (SSS), and supraventricular tachycardia (SVT).

**Results:**

A total of 25,438 patients were included in each cohort after matching. Compared to general population, the IE cohort had significantly higher 5-year cumulative incidence of AF (3.6% vs 0.7%; HR, 6.14; 95% CI, 5.25–7.19), VF (0.2% vs < 0.1%; HR, 8.63), complete AV block (0.5% vs < 0.1%; HR, 16.74), SSS (0.6% vs 0.1%; HR, 11.59), and SVT (1.1% vs 0.3%; HR, 4.51) (all *P*< 0.001, Figure 1). Kaplan-Meier curves showed early separation between groups that persisted throughout follow-up.

**Conclusion:**

In this large, real-world cohort study, infective endocarditis was associated with significantly elevated risk of multiple arrhythmia subtypes over 5 years. These findings support long-term cardiac monitoring and follow-up strategies in patients recovering from IE.

**Disclosures:**

All Authors: No reported disclosures

